# Current management of biliary atresia based on 35 years of experience at a single center

**DOI:** 10.6061/clinics/2018/e289

**Published:** 2018-06-29

**Authors:** Wagner de Castro Andrade, Marcos Marques Silva, Ana Cristina Aoun Tannuri, Maria Merces Santos, Nelson Elias Mendes Gibelli, Uenis Tannuri

**Affiliations:** Divisao de Cirurgia Pediatrica, Unidade Pediatrica de Transplante de Figado e Laboratorio de Pesquisa em Cirurgia Pediatrica (LIM 30), Faculdade de Medicina FMUSP, Universidade de Sao Paulo, Sao Paulo, SP, BR

**Keywords:** Biliary Atresia/Surgery, Hepatic Portoenterostomy, Survival Rate, Liver Transplantation, Neonatal Jaundice

## Abstract

**OBJECTIVE:**

The prognosis of patients with biliary atresia undergoing Kasai portoenterostomy is related to the timing of the diagnosis and the indication for the procedure. The purpose of the present study is to present a practical flowchart based on 257 children who underwent Kasai portoenterostomy.

**METHODS:**

We conducted a retrospective cohort study of patients who underwent Kasai portoenterostomy between 1981 and 2016.

**RESULTS:**

During the first period (1981 to 2009), 230 infants were treated, and the median age at the time of surgery was 84 days; jaundice was resolved in 77 patients (33.5%). During the second period, from 2010 to 2016, a new diagnostic approach was adopted to shorten the wait time for portoenterostomy; an ultrasonography examination suggestive of the disease was followed by primary surgical exploration of the biliary tract without complementary examination or liver biopsy. Once the diagnosis of biliary atresia was confirmed, a portoenterostomy was performed during the same surgery. During this period, 27 infants underwent operations; the median age at the time of surgery was 66 days (*p*<0.001), and jaundice was resolved in 15 patients (55.6% - *p*=0.021), with a survival rate of the native liver of 66.7%.

**CONCLUSION:**

Primary surgical exploration of the biliary tract without previous biopsy was effective at improving the prognostic indicators of patients with biliary atresia undergoing Kasai portoenterostomy.

## INTRODUCTION

Biliary atresia (BA) is a rare disease of infancy, and patients present with jaundice and pale stools in the first few weeks of life. The etiopathogenesis of BA is not fully understood, and the disease is likely the phenotypic manifestation of various mechanisms that can act alone or jointly. Genetic causes, exogenous agents (viruses and toxins), immunological mechanisms and ductal plaque malformations have been implicated in the development of this condition. If left untreated, BA rapidly evolves into liver failure secondary to biliary cirrhosis, and the mean survival rate is less than 2 years 1,2. Histologically, BA is characterized as an obliterative cholangiopathy. In most cases, it obliterates the luminal opening of the most proximal part of the extrahepatic biliary tree. Kasai portoenterostomy (KP), considered the standard surgical treatment for this condition, restores bile flow to the intestinal lumen through residual ductules in the *porta hepatis* region that retain some connection to the potentially preserved intrahepatic biliary tree 3. Although KP is successful in many infants, the procedure has a high failure rate. Some patients receive no benefit from the procedure and eventually develop end-stage liver disease. For these patients, the only option is liver transplantation (LT). In Brazil, BA is the indication for approximately 60% of LTs 4,5. Our center conducted the first pediatric LT in Brazil (1989), and more than 700 procedures have been performed since.

In 1999, the first national survey on BA was conducted, compiling 664 cases from 18 Brazilian institutions (non-published data). The median age of the children who underwent KP was 90 days, which is much older than that reported in other centers worldwide 6-8. Since the survey was conducted, an effort has been made by public health services and physicians to more quickly refer suspected patients to specialized centers, although at present, Brazil lacks the clear and rigorous regionalization found in the UK 9 and other countries. Aiming to shorten the time that patients wait for a portoenterostomy, our hospital adopted a new diagnostic approach for suspected cases of BA in 2010. In cases with a high clinical index of suspicion (progressive cholestasis, persistent acholic stools) supported by an ultrasonography examination that excludes other possible surgical diagnoses (e.g., choledochal malformation, inspissated bile syndrome, bile duct perforation) and shows an atrophic gallbladder or the triangular cord sign, we perform primary surgical exploration of the bile ducts through a small right subcostal laparotomy without previous complementary examinations or liver biopsy. Once the diagnosis of BA is confirmed (occasionally with intraoperative cholangiography), we extend the incision and perform a portoenterostomy during the same surgery. The aim of this study is to report the results of BA surgery performed between 1981 and 2016 (257 patients) at a Brazilian medical center and to analyze the impact of primary surgical exploration without previous liver biopsy on the clearance of jaundice.

## PATIENTS AND METHODS

**Figure 1:**
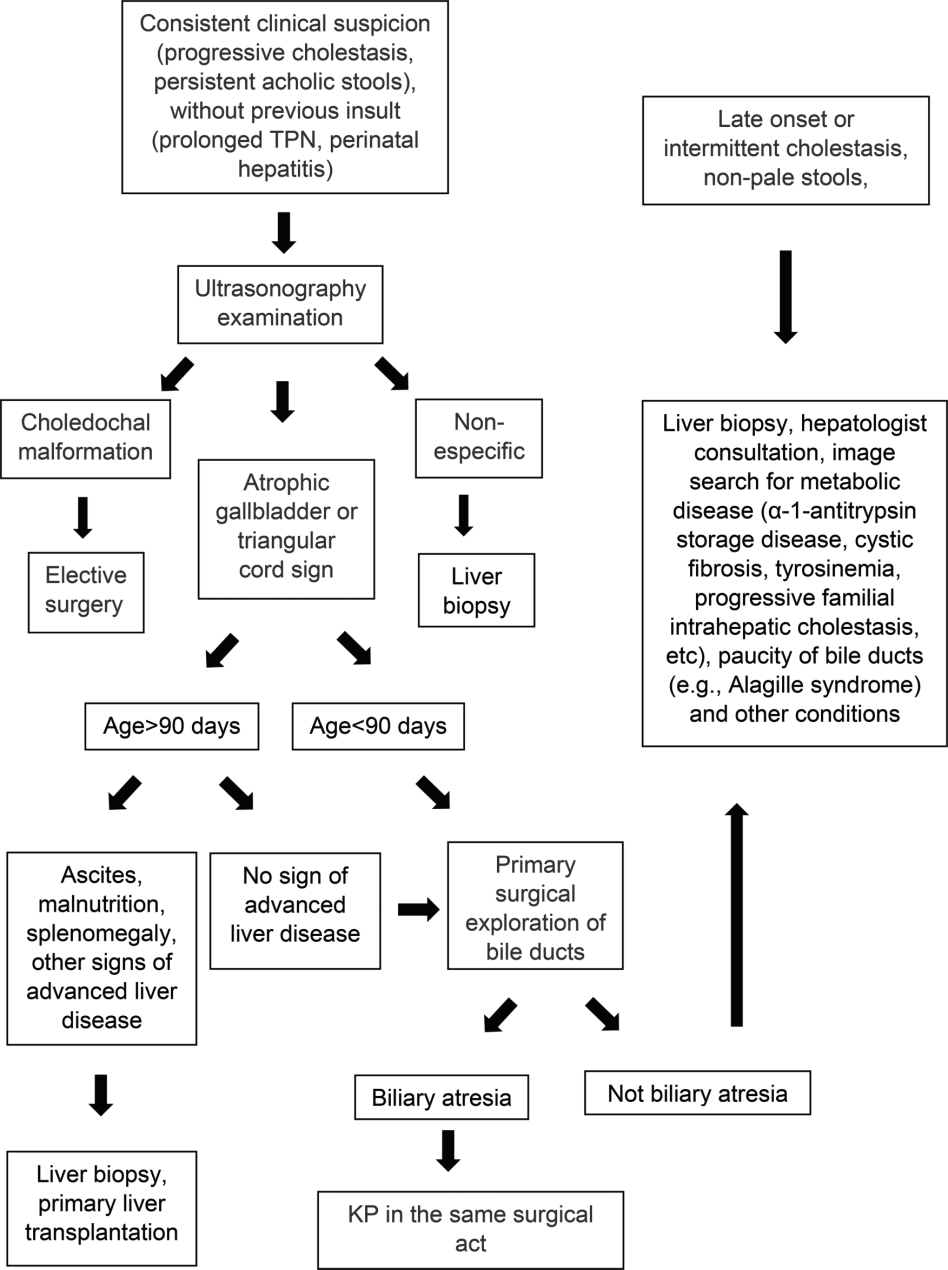
Perinatal cholestasis flowchart (TPN – total parenteral nutrition; KP – Kasai portoenterostomy).

The study was approved by the Ethics Committee for Research Project Analysis at our institution. The records of all consecutive patients undergoing Kasai surgery at our institution between 1981 and 2009 were retrospectively reviewed. From 2010 onward, a second group of consecutive patients with suspected BA were managed according to a new protocol aimed at shortening the wait time for portoenterostomy (Figure 1).

**Figure 2:**
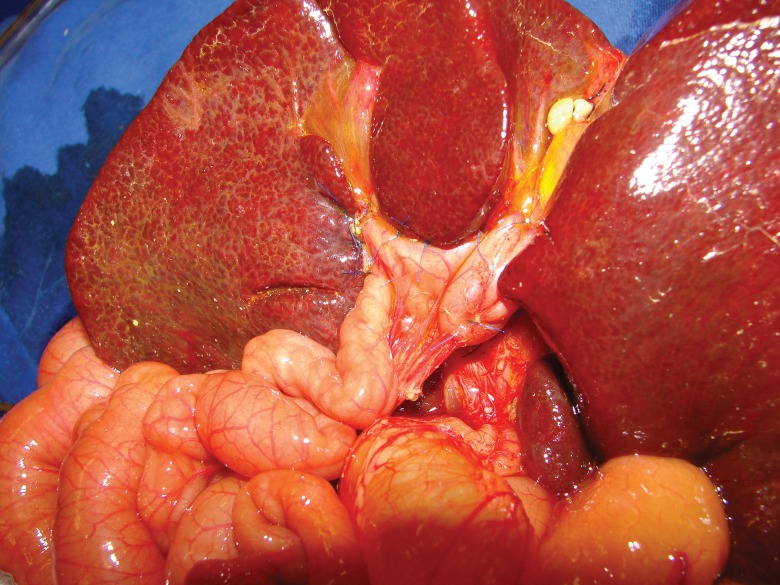
Final aspect of the surgery. Note the Roux-en-Y jejunal loop draining the transected *porta hepatis*.

All patients were operated on by the same group of surgeons at our institution. Infants underwent surgical excision of the biliary remnant with resection extended to the border of the portal vein bifurcation according to the principles described by Kasai 3. Remnant excision was followed by a 40-cm Roux-en-Y loop biliary reconstruction (Figure 2). During the early period, 33 infants had an initial but temporary stoma constructed in the Roux loop (Suruga II modification). We did not adopt the laparoscopic approach because there is no evidence to support its superiority or even equivalence in the later results 10. The diagnosis of BA was confirmed in all cases by histological analysis of the liver and biliary remnants. We included the 257 patients who underwent operations during the last 35 years (mean of 7 cases/year) and received regular follow-up for at least 12 months in our care.

Postoperative care has evolved over time and has included ursodeoxycholic acid and oral antibiotics since the 1990s. Steroids (1 mg/kg/day prednisone for 4 weeks; therapy is initiated after the postoperative fasting period, followed by a 4-week taper) have been used since 2007. All patients were followed-up for a minimum of 12 months.

Comparisons of the data were performed with IBM SPSS Statistics 17 (SPSS Inc. Released 2008. SPSS Statistics for Windows, Version 17.0. Chicago, IL, USA: SPSS Inc.). We compared the data from the two study periods to verify the effect of the new diagnostic and therapeutic approach. Categorical variables were compared by *χ^2^* tests. Native liver survival was expressed as a Kaplan-Meier curve, with both LT and death treated as events. *p*-values less than 0.05 were regarded as significant.

## RESULTS

The demographic data of patients are included in Table 1. Among all BA patients, microscopic examination of the transected bile duct remnant revealed 23.9% type I, 30.4% type II and 45.7% type III portal plates. Eight patients were identified as having an associated splenic malformation (polysplenia with or without a preduodenal portal vein).

During the first period, 230 patients were treated. The median age at KP was 84 days (11-188 days). Jaundice was resolved in 77 patients (33.5%) within 3 months of portoenterostomy. During the second period, 27 infants with BA underwent surgery. The median age at KP was 66 days (28-103 days), and the mean body weight was 4.97±0.38 Kg, both of which were significantly different from the previous period (*p*<0.001). Jaundice was resolved in 15 patients (55.6%) during the second period, which was a significant increase compared to the first period (*χ^2^*=5.354; *p*=0.021).

**Figure 3:**
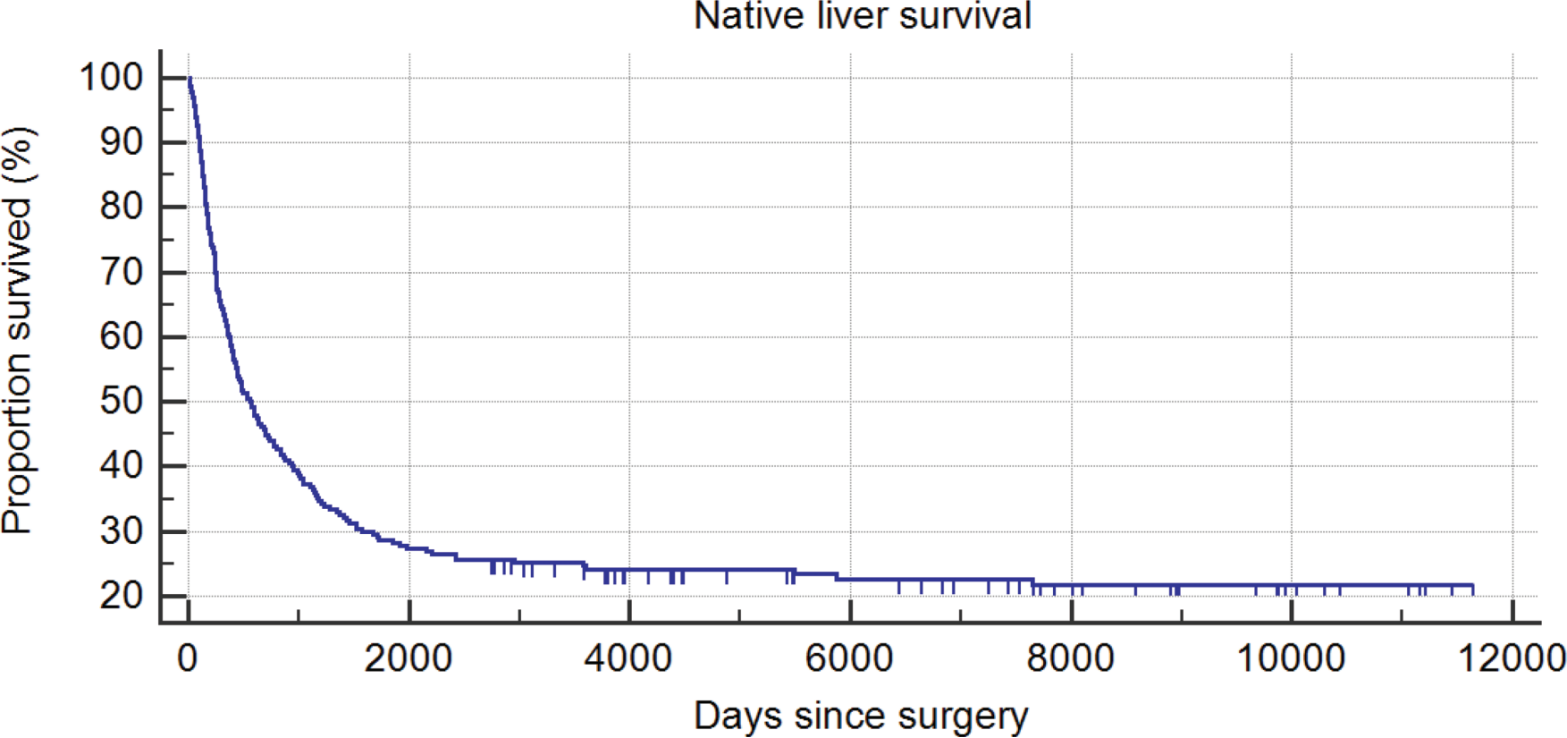
Kaplan-Meier native liver survival curve (1981-2009 / n=230).

**Figure 4:**
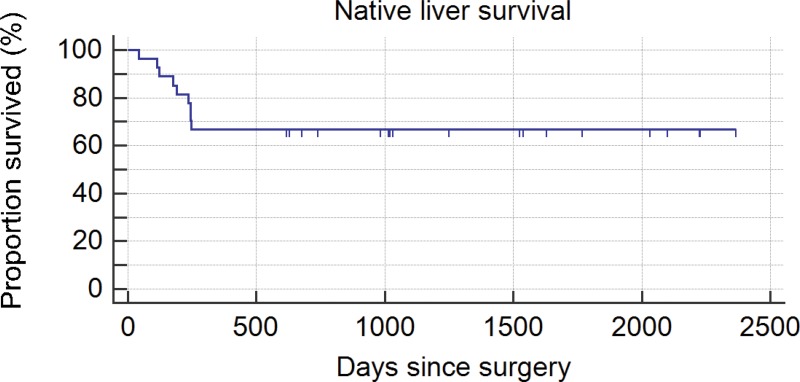
Kaplan-Meier native liver survival curve (2010-2016 / n=27).

Among the anicteric patients in the first period, 19 (24.7%) later underwent LT (more than 2 years after KP) due to recurrent cholangitis and a relapse of jaundice, hepatic dysfunction, uncontrollable portal hypertension or hepatopulmonary syndrome. During the second period, among all KP patients, five (18.5% of total) underwent LT, and four icteric patients died while on the waiting list for LT due to sepsis or hemorrhage (due to esophageal varices). Hepatopulmonary syndrome developed in one jaundice-free child at one year after surgery. The survival rate of the native liver in the second group was higher than that in the first group (Figures 3 and 4). The mean follow-up through the end of the study in this group was 1903.2 days. Twenty-nine patients from the first period and 2 patients from the second period were excluded due to a lack of information during follow-up.

Exploration of the bile ducts without previous liver biopsy followed by optional KP during the same surgery characterized the strategy used to manage patients who were treated during the second period. Using this new protocol, 2 patients underwent surgical exploration despite an intraoperative finding of patent bile ducts. In each case, we performed a liver biopsy. The histopathological diagnoses were neonatal hepatitis in one case and ductopenia in the other.

## DISCUSSION

Biliary atresia is a rare disease, with estimates of its prevalence ranging from 1 in 6,000 in Taiwan to 1 in 20,000 in Northern Europe 8,11. Therefore, the current birth rate in Brazil (approximately 3,000,000 annually for the last 5 years) suggests that 150 to 300 infants with BA are born each year in our country.

The currently accepted standard treatment for BA is a KP performed at the earliest possible age by experienced surgeons. LT is indicated for children in whom jaundice does not resolve after KP and who therefore develop end-stage liver disease or complications. Transplantation should be performed as timely as possible to avoid death of patients on the waiting list.

Although variation has been observed in the postoperative evolution in children after Kasai’s surgery 12, the median time to KP is likely a relevant factor in the prognosis. This analysis allows the inference of a population’s access to basic health services and the diagnostic efficiency of each region. In this cohort, we identified a significant evolution in our institution over time, characterized by significant improvement in surgical outcomes, particularly during the last decade. Despite this success, a lack of investment in primary care, poor resource management and great variability in access to specialized resources represent substantial challenges to our ability to care for these patients. Brazil is a country of continental dimensions that does not yet have well-structured regional or central medical systems.

To minimize the interval between diagnostic suspicion and surgical treatment, we chose to dispense with the traditional time-consuming complementary tests (endoscopic retrograde cholangiopancreatography, radio-isotope-labeled hepatobiliary scans, and even percutaneous liver biopsy) in most cases presenting with strong clinical/ultrasonographic evidence of BA. We performed open surgical exploration of the biliary tract with an optional intraoperative cholangiography when BA type 1 or 2 was suspected. We believe that this protocol should be generally adopted and that it can be a simple and effective alternative that reduces the median time to KP, especially in countries with geographic, demographic and economic characteristics similar to Brazil.

In two cases, we performed a laparotomy even though BA was not confirmed. In our view, no increase in morbidity is caused by performing a small right subcostal laparotomy with biopsy and cauterization of the liver capsule, which minimizes the risk of bleeding. Another possible alternative was initial laparoscopy for all patients, but since the definitive treatment of confirmed BA cases would necessitate an open incision, we did not adopt this strategy. Finally, it is important to emphasize that although laparoscopic portoenterostomy represents a tempting procedure for the correction of BA, outcomes in terms of native liver survival rates and actuarial survival rates are not superior to those of conventional surgery by open laparotomy, as we propose herein. There is no evidence showing that laparoscopic KP performed prior to a subsequent LT is associated with fewer adhesions 10. Finally, our results suggest that laparoscopic KP may be abandoned in pediatric surgery.

We were pleased to identify a dramatic improvement in the percentage of jaundice clearance (55.6%) in recent years. In addition, our recent actuarial native liver survival (66.7%) and patient survival (77.8%) rates are comparable to those of other large studies 12.

In conclusion, the current study shows that the measures undertaken in recent years, including primary surgical exploration of the bile tract without previous biopsy, have resulted in significant improvements in two key indices commonly used to characterize a BA program (median time to KP and jaundice clearance).

## AUTHOR CONTRIBUTIONS

Andrade WC and Silva MM conceived and designed the study and were responsible for the interpretation of data and manuscript drafting. Tannuri AC, Santos MM and Gibelli NE were responsible for the acquisition and analysis of data. Tannuri U was responsible for the final interpretation of data and critical revision.

## Figures and Tables

**Table 1:** Demographic data of patients from the two study groups.

		1981 - 2009	2010 - 2016
Gender	Male	90 (39.1%)	12 (44.4%)
	Female	140 (60.9%)	15 (55.6%)
Weight kg (Mean±sd)		5.34±0.43	4.97±0.38*

* *p*<0.001.
